# Supplementary material for: The Physicochemical Characteristics and Heavy Metal Retention Capability of Black Liquor Lignin-Based Biochars
Source: Molecules. 2023 Nov 21;28(23):7694. doi: 10.3390/molecules28237694 (PMC10708106; doi:10.3390/molecules28237694)
Supplement: Supplementary file 1 [file molecules-28-07694-s001.zip › molecules-2688801-supplementary.pdf]

Supplementary materials

for

Characteristics and Cd(II) adsorption capability of black liquor lignin-based biochars: Effect of pyrolysis temperature

1 Data analysis

1.1 Adsorption isotherm model analysis

Adsorption isotherms were analyzed using the Langmuir model and the Freundlich model to explore how the adsorbate interacts with the adsorbent [1]. Among them, the calculation equation of the Langmuir model is:

$$Q_e = \frac{Q_m K_l C_e}{1 + K_l C_e} \quad \text{Eq. S1}$$

The calculation equation of the Freundlich model is:

$$Q_e = K_f C_e^{1/n} \quad \text{Eq. S2}$$

where, Q_e is the equilibrium adsorption capacity of Cd(II) by LGBCs (mg/g); C_e is the equilibrium concentration of Cd(II) solution after adsorption by LGBCs (mg/L); Q_m is the theoretical maximum adsorption capacity (mg/g); K_l is the Langmuir adsorption constant (L/mg) related to the interaction energy; K_f is the Freundlich affinity coefficient ($(mg^{(1-n)}L^n)/g$); n is the Freundlich intensity constant.

1.2 Adsorption kinetic model analysis

The adsorption kinetics were explained using pseudo-first-order kinetics and pseudo-second-order kinetics. Among them, the pseudo-first-order kinetics mainly describe the adsorption rate in the liquid system [2], and the calculation equation of this model is:

$$\frac{dQ_t}{dt} = k_1(Q_{e,the} - Q_t) \quad \text{Eq. S3}$$

The pseudo-second-order kinetics is used to describe the chemisorption between metal ions and adsorption sites of the adsorbent during the adsorption process, and the calculation equation of this model is:

$$\frac{dQ_t}{dt} = k_2(Q_{e,the} - Q_t)^2 \quad \text{Eq. S4}$$

where, t is the reaction time (h); Q_t is the adsorption amount of Cd(II) by LGBCs at the reaction time point t (mg/g); $Q_{e,\,the}$ is the calculated equilibrium adsorption amount of Cd(II) by LGBCs (mg) /g); k_1 is the adsorption rate constant (h^{-1}); k_2 is the adsorption rate constant (g/(mg·h)).

1.3 Thermodynamic analysis

The adsorption thermodynamic model is a crucial to describe the metal adsorption process and explore the mechanism, which can be characterized by three parameters regarding to Gibbs free energy (ΔG^0), enthalpy (ΔH^0), and entropy (ΔS^0).

$$\Delta G^0 = -RT \ln K_e \qquad \text{Eq.S5}$$

K_e needs to be calculated from the partition constant (K_w) and the density of the solvent (ρ):

$$K_\omega = \frac{Q_e}{C_e} \qquad \text{Eq.S6}$$

$$K_e = \rho K_\omega = \rho \frac{Q_e}{C_e} \qquad \text{Eq.S7}$$

Since the density of water is 1 g/cm^3, Equation Eq.S7 can be written as:

$$K_e = \frac{Q_e}{C_e} \qquad \text{Eq.S8}$$

During the reaction process, ΔG^0, ΔH^0 and ΔS^0 have the following equations:

$$\Delta G^0 = \Delta H^0 - T\Delta S^0 \qquad \text{Eq.S9}$$

Combined with Equation (13), we get:

$$\ln K_e = -\frac{\Delta H^0}{RT} + \frac{\Delta S^0}{R} \qquad \text{Eq.S10}$$

where, K_e is the dimensionless adsorption equilibrium constant; R is the gas molar constant (8.314 J/(mol·K)); T is the absolute temperature (K); Q_e is the equilibrium adsorption amount of Cd(II) by LGBCs (mg/g); C_e is the equilibrium concentration (mg/L) after adsorption of Cd(II) solution by LGBCs; ρ is the density of water (g/cm^3).

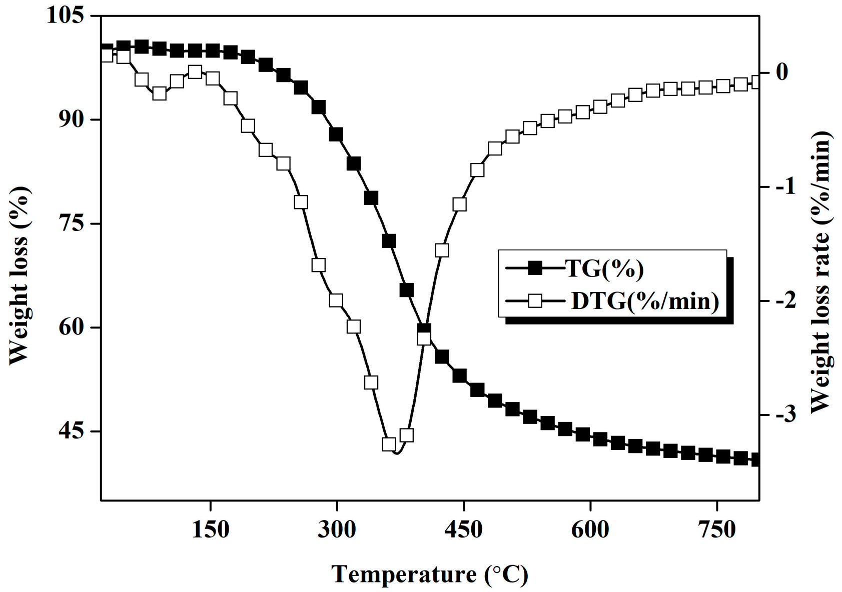

Figure S1. TG and DTG curves of LG

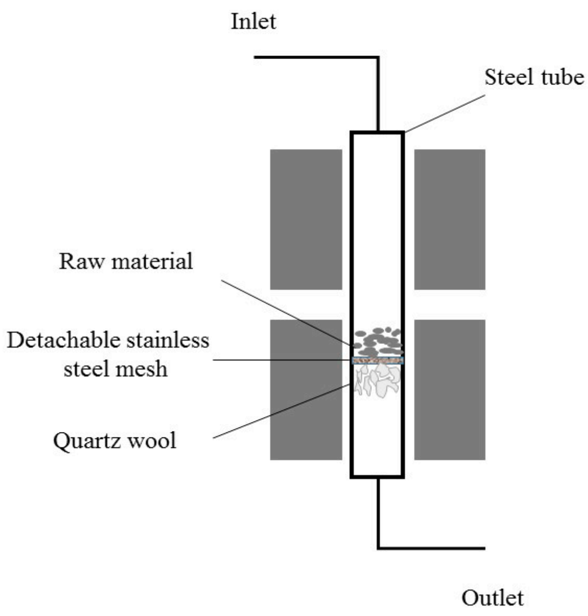

Figure S2. Experimental setup

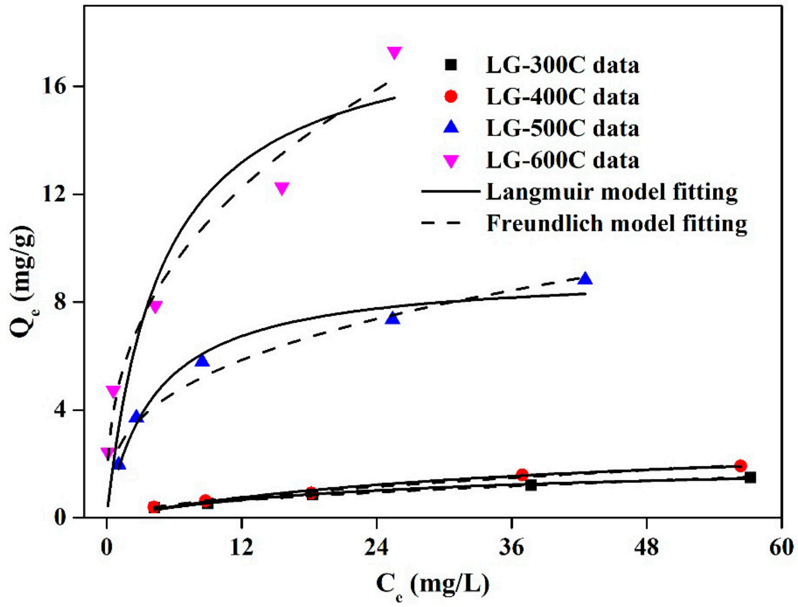

Figure S3. Adsorption isotherm fitting of Cd(II) adsorption on LGBCs (C_e and Q_e are the equilibrium concentration of Cd(II) after adsorption and adsorption capacity of Cd(II) by LGBCs, respectively). The initial concentration of Cd(II), contact time, ambient temperature, and solution pH were set as follows: 5~60 mg/L, 8 h, 25 ℃, and 5, respectively.

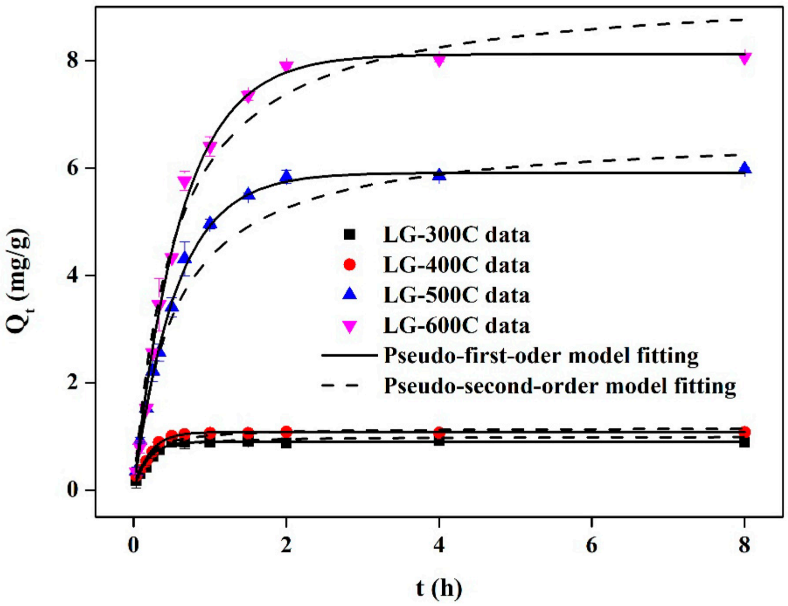

Figure S4. Adsorption kinetics fitting of Cd(II) adsorption on LGBCs (t and Qt are contact time and the adsorption capacity at contact time t, respectively). The initial concentration of Cd(II), contact time, ambient temperature, and solution pH were set as follows: 20 mg/L, 2 min~8 h, 25 ℃, and 5, respectively.

Table S1 Physicochemical properties of LG

Elemental analysis				Proximate analysis			Other	
C	H	O	S	Fixed carbon	Volatile matter	Ash	pH	Specific surface area
%				%				m^2/g
59.62	5.74	30.14	1.69	27.41	67.52	2.81	5.62	1.13

References:

1. Qin, K.; Li, J.; Yang, W.; Wang, Z.; Zhang, H., Role of minerals in mushroom residue on its adsorption capability to Cd(II) from aqueous solution. *Chemosphere* **2023,** 324, 138290.
2. Kılıç, M.; Kırbıyık, Ç.; Çepelioğullar, Ö.; Pütün, A. E., Adsorption of heavy metal ions from aqueous solutions by bio-char, a by-product of pyrolysis. *Applied Surface Science* **2013,** 283, (0), 856-862.
